# Supplementary material for: Humans use forward thinking to exploit social controllability
Source: eLife. 2021 Oct 29;10:e64983. doi: 10.7554/eLife.64983 (PMC8555988; doi:10.7554/eLife.64983)
Supplement: Supplementary file 1. [file elife-64983-supp1.docx]

**Supplementary Information**

**Humans Use Forward Thinking to Exploit Social Controllability**

Soojung Na*, Dongil Chung*, Andreas Hula, Ofer Perl, Jennifer Jung, Matthew Heflin, Sylvia Blackmore, Vincenzo G. Fiore, Peter Dayan, and Xiaosi Gu

**Supplementary file 1a.** To further explore whether emotional responses differed by the task type, we ran a mixed effect regression model (emotion rating ~ offer + norm prediction error + condition + task + task*(offer + norm prediction error + condition) + (1 + offer + norm prediction error | subject)). There was a significant interaction effect of the norm prediction error and the task in which the impacts of norm prediction errors on emotion ratings were significantly greater for the social task than for the non-social task.

| Name | Estimate | SE | T | DF | P-value |
| --- | --- | --- | --- | --- | --- |
| Intercept (***) | 31.25 | 3.83 | 8.15 | 2789 | 0.000 |
| Norm prediction error (nPE) | 0.12 | 0.26 | 0.47 | 2789 | 0.642 |
| Offer (***) | 3.34 | 0.56 | 5.92 | 2789 | 0.000 |
| Social task | -0.19 | 2.39 | -0.08 | 2789 | 0.936 |
| Controllable | 0.62 | 1.23 | 0.51 | 2789 | 0.613 |
| nPE × social task (*) | 0.52 | 0.26 | 1.96 | 2789 | 0.050 |
| Offer × social task | 0.71 | 0.44 | 1.62 | 2789 | 0.106 |
| Controllable × social task (***) | -5.06 | 1.47 | -3.45 | 2789 | 0.001 |

* *P* < .05. *** *P* < .001

**Supplementary file 1b.** We ran a mixed effect generalized linear model (choice ~ 1 + offer + condition + offer*condition + (1 + offer + condition + offer*condition | subject) to test whether the results from the simple analysis held even after controlling for the varying individual random effects. We found a significant offer effect and a marginal interaction effect of the condition and the offer, whereas the condition did not have a significant effect on choices. These results further corroborate our simple analysis results that mean rejection rates were comparable between the conditions (**Figure 2b1**) and that rejection rates decreased as offer increased with a different slope by condition (**Figure 2b2)**.

| Name | Estimate | SE | t | DF | p-value |
| --- | --- | --- | --- | --- | --- |
| Intercept (***) | -8.61 | 0.71 | -12.09 | 3792 | 0.000 |
| Condition | -0.58 | 1.02 | -0.57 | 3792 | 0.568 |
| Offer (***) | 1.82 | 0.14 | 13.27 | 3792 | 0.000 |
| Condition × Offer | -0.30 | 0.18 | -1.65 | 3792 | 0.099 |

The Controllable condition was coded as 1 and the Uncontrollable condition was coded as 0; *** *P* < 0.001

**Supplementary file 1c.** We ran a mixed effect generalized linear model (RT ~ 1+ condition + chosen values + condition * chosen values + (1+ chosen values | subject)) using log as the link function to test whether chosen values predict response times. We found that neither the chosen value coefficient (beta = 0.00, *P* = .95) nor the interaction term (beta = -0.00, *P* = .73) was significant, while the condition effect was significant (beta = 0.11, *P* < .001; consistent with **Figure 2d**). *** *P* < 0.001

| Name | Estimate | SE | t | DF | p-value |
| --- | --- | --- | --- | --- | --- |
| Intercept | 0.41 | 0.04 | 9.79 | 2860 | 0.000 |
| Condition (***) | 0.11 | 0.03 | 3.35 | 2860 | 0.001 |
| Chosen value | 0.00 | 0.00 | 0.07 | 2860 | 0.945 |
| Condition × chosen value | 0.00 | 0.00 | -0.34 | 2860 | 0.737 |

*** *P* < 0.001

**Supplementary file 1d.** We ran a mixed effect generalized linear model (RT ~ 1+ condition + conflict + condition * conflict + (1+ conflict | subject)) using log as the link function to test whether conflicts (values of the chosen action – values of the unchosen action) affect response times. Both the conflict effect (beta = -0.03, *P* < .001) and the interaction effect between the condition and conflict (beta = 0.02, *P* < .05) were significant as well as the condition effect (beta = 0.08, *P* < .001), suggesting that conflict did have a significant impact on RT. * *P* < 0.05; *** *P* < 001.

| Name | Estimate | SE | t | DF | p-value |
| --- | --- | --- | --- | --- | --- |
| Intercept | 0.45 | 0.04 | 12.29 | 2860 | 0.000 |
| Condition (***) | 0.08 | 0.02 | 4.45 | 2860 | 0.000 |
| Conflict (***) | -0.03 | 0.01 | -3.31 | 2860 | 0.001 |
| Condition × conflict (*) | 0.02 | 0.01 | 2.21 | 2860 | 0.027 |

** *P* < 0.01; *** *P* < 001.

**Supplementary file 1e. Positive effect of total chosen values (both current and future) from the 2-step FT model (Controllable and Uncontrollable combined)**

| Region | Lat | x | y | z | T | Z | k |
| --- | --- | --- | --- | --- | --- | --- | --- |
| Postcentral gyrus | R | 42 | -22 | 50 | 6.32 | 5.35 | 1004 |
| Cerebellum | L | -26 | -84 | -20 | 4.6 | 4.16 | 233 |
| vmPFC | L | 0 | 54 | -2 | 4.57 | 4.14 | 147 |
| Cerebellum | L | -32 | -60 | -22 | 4.38 | 3.99 | 249 |
| Superior parietal lobule | R | 28 | -56 | 66 | 4.05 | 3.73 | 51 |

(*P_FDR_* < 0.05 and *k* > 50)

**Supplementary file 1f. Positive effect of total chosen values (current value only) from the 0-step no planning model (Controllable and Uncontrollable combined).** No significant vmPFC activation at *P* < 0.005 uncorrected and *k* > 50.

| Region | Lat | x | y | z | T | Z | k |
| --- | --- | --- | --- | --- | --- | --- | --- |
| Postcentral gyrus | R | 42 | -22 | 48 | 4.27 | 3.9 | 841 |
| Cerebellum | L | -16 | -54 | -20 | 3.57 | 3.34 | 135 |
| Inferior temporal gyrus | R | 58 | -52 | -6 | 3.36 | 3.16 | 70 |

(*P* < 0.005 uncorrected and *k* > 50)

**Supplementary file 1g. Uncertainty and autocorrelation effects**

**i.** Expected influence ~ 1 + offer SD + condition

| Name | Estimate | SE | tStat | DF | pValue | Lower | Upper |
| --- | --- | --- | --- | --- | --- | --- | --- |
| '(Intercept)' | 1.01 | 0.39 | 2.59 | 93 | 0.011 | 0.24 | 1.79 |
| Condition | 0.36 | 0.15 | 2.45 | 93 | 0.016 | 0.07 | 0.64 |
| Offer SD | -0.03 | 0.32 | -0.10 | 93 | 0.922 | -0.66 | 0.60 |

**ii.** Self-reported perceived controllability ~ 1 + offer SD + condition

| Name | Estimate | SE | tStat | DF | pValue | Lower | Upper |
| --- | --- | --- | --- | --- | --- | --- | --- |
| '(Intercept)' | 25.00 | 13.83 | 1.81 | 83 | 0.074 | -2.51 | 52.51 |
| Condition | 21.09 | 5.31 | 3.97 | 83 | 0.000 | 10.52 | 31.65 |
| Offer SD | 15.60 | 11.11 | 1.40 | 83 | 0.164 | -6.50 | 37.69 |

**iii.** Expected influence ~ 1 + ACF1 + condition

| Name | Estimate | SE | tStat | DF | pValue | Lower | Upper |
| --- | --- | --- | --- | --- | --- | --- | --- |
| '(Intercept)' | 0.95 | 0.11 | 8.68 | 93 | 0.000 | 0.74 | 1.17 |
| Condition | 0.46 | 0.25 | 1.85 | 93 | 0.067 | -0.03 | 0.95 |
| ACF1 | -0.21 | 0.39 | -0.53 | 93 | 0.599 | -0.99 | 0.57 |

**iv.** Self-reported perceived controllability ~ 1 + ACF1 + condition

| Name | Estimate | SE | tStat | DF | pValue | Lower | Upper |
| --- | --- | --- | --- | --- | --- | --- | --- |
| '(Intercept)' | 44.50 | 4.07 | 10.93 | 83 | 0.000 | 36.40 | 52.60 |
| Condition | 18.14 | 9.17 | 1.98 | 83 | 0.051 | -0.10 | 36.37 |
| ACF1 | 7.93 | 14.54 | 0.55 | 83 | 0.587 | -20.99 | 36.85 |

**Supplementary file 1h. Order effects**

|  | PC_C_ | PC_U_ | Delta_C_ | Delta_U_ |
| --- | --- | --- | --- | --- |
| Group1 (Controllable first) | 64.76 | 45.43 | 1.28 | 0.95 |
| Group2 (Uncontrollable first) | 66.96 | 41.58 | 1.38 | 1.00 |
| df | 20 | 18 | 23 | 23 |
| *t* | -0.94 | 0.96 | -0.44 | -0.34 |
| *P* | 0.36 | 0.35 | 0.66 | 0.74 |

Although we did not find any order effect on the expected influence parameter or self-reported belief, future studies would be needed to probe task-induced priors more thoroughly. PC represents self-reported perceived controllability. C represents the Controllable condition and U represents the Uncontrollable condition.
