# Supplementary material for: Humans use forward thinking to exploit social controllability
Source: eLife. 2021 Oct 29;10:e64983. doi: 10.7554/eLife.64983 (PMC8555988; doi:10.7554/eLife.64983)
Supplement: Supplementary file 2. [file elife-64983-supp2.docx]

**Supplementary file 2. Task instructions.** We provided participants with the written instructions below:

| Task Instructions (Compensation $0-10)  In this task you will participate in a series of decisions to split $20.00 with a partner. Your partner will propose how to split the money. **You will decide whether or not to accept the proposal.** If you accept the proposal, you will each get the share of the split your partner proposed. If you reject the proposal, however, both of you will get nothing.  At the beginning of each round you will be randomly paired up with a **new** partner. Because this is done randomly, you will be playing with a different partner in each round. You will not meet any of your partners at any point throughout the session.  You will play with two groups in a random order and each group has 40 players.  **Here is what the game will look like:**  You will be assigned a group of proposers to play the game with.  You will be playing with proposers from Group A  **HIT SPACE TO CONTINUE**  At the beginning of each round, you will be randomly paired with a partner and you will see their proposal to split money between you and your partner:  H.T. proposes you: $6  You can choose to accept the proposed split or reject the offer.  H.T. YOU  $14 $6  <- ACCEPT REJECT->  You receive $6  After some trials you will be asked how you feel about the proposal. Use left and right arrow keys to move the slider bar. To submit your choice, press the spacebar.  How do you feel?    Bad Good  Press Space bar to confirm  If you reject the offer, both you and your partner will not earn any money.  After you see the result of the proposal, you will be randomly paired with a new partner and a new round will begin. You will play **80** rounds of this game in total.  BONUS PAYMENT: At the end of the experiment the computer will randomly draw one of the rounds you played and pay you a bonus according to the outcome of that round: your share of the money if your partner accepted the offer, or nothing if they rejected the offer. Because any **one** of the choices could count “for real”, we encourage you to make each choice as though it is the one you are actually going to receive. |
| --- |
